# In-Situ Monitoring of Machining Loads and Cross-Scale Characterization of Surface Integrity and Electrochemical Response During Ultrasonic-Assisted Milling of Ti-6Al-4V

**DOI:** 10.3390/ma19183850

**Published:** 2026-09-10

**Authors:** Qian Qiao, Dawei Guo, Chi-Tat Kwok, Lap-Mou Tam

**Affiliations:** 1 IDQ Science and Technology (Hengqin, Guangdong) Co., Ltd., Zhuhai 519000, China; qqiao@idq.org.mo; 2 Institute for the Development and Quality, University of Macau, Macao 999078, China; 3 Department of Electromechanical Engineering, University of Macau, Macao 999078, China; fstctk@um.edu.mo

**Keywords:** ultrasonic vibration-assisted machining, Ti6Al4V, in-situ monitoring, microstructure, corrosion

## Abstract

Ultrasonic vibration-assisted machining (UVAM) can improve the machinability of difficult-to-machine titanium alloys; however, the relationship between machining-load fluctuations, subsurface microstructure, and electrochemical response remains insufficiently established. In this study, a wireless in-situ monitoring system was integrated with electron backscatter diffraction (EBSD), electrochemical impedance spectroscopy (EIS), potentiodynamic polarization, and scanning electrochemical microscopy (SECM) to compare conventional milling (CM), low-excitation UVAM (L-UVAM), and high-excitation UVAM (H-UVAM) of Ti-6Al-4V. Under the investigated conditions, H-UVAM reduced the RMS value of the measured axial load signal by 43.3% compared with CM and decreased the variation in the resultant bending-moment signal. The EBSD results showed a reduction in the mean grain size from 11.67 μm for CM to 10.07 μm for H-UVAM, together with an increase in the measured high-angle grain-boundary fraction from 48.27% to 59.39%. Electrochemical measurements further indicated a lower corrosion current density and a higher fitted barrier resistance for the H-UVAM surface. SECM mapping showed a narrower local current distribution under H-UVAM than under CM. These results demonstrate a consistent association between reduced machining-load fluctuations, modified subsurface crystallographic features, and improved electrochemical response. Because surface roughness, residual stress, tool wear, and passive-film chemistry were not independently quantified, the present work does not attribute the corrosion response exclusively to microstructural changes. Instead, it provides a cross-scale experimental framework for correlating machining dynamics with surface integrity and corrosion-related performance in machined titanium alloys.

## 1. Introduction

Owing to their exceptional specific strength, superior heat resistance, and inherent macroscopic anti-corrosion properties, titanium alloys (particularly Ti6Al4V) have been unequivocally established as indispensable structural materials in critical aerospace and biomedical engineering sectors [1,2]. Nevertheless, the intrinsic thermomechanical characteristics of titanium—specifically its low thermal conductivity coupled with high chemical reactivity at elevated temperatures—render it a notoriously difficult-to-machine material. During the conventional milling process, the persistent generation of localized thermal concentration provokes rapid tool degradation [3], profound surface mechanical deterioration, and the undesirable induction of residual tensile stresses, which collectively compromise the fatigue life and functional integrity of the machined components [4,5].

To mitigate these profound machinability bottlenecks, Ultrasonic Vibration-Assisted Machining (UVAM) has emerged as a disruptive non-traditional manufacturing paradigm [6,7]. Prevailing theoretical frameworks, heavily substantiated by preliminary observations in ultrasonic-assisted turning processes, posit that the superimposition of high-frequency acoustic vibrations fundamentally alters the continuous tool–workpiece engagement [8,9]. This modulation induces a characteristic kinematic separation phenomenon, effectively minimizing the effective tool-cutting duration per cycle. Consequently, this intermittent cutting behavior facilitates superior coolant penetration and chip evacuation, translating into significantly attenuated average cutting forces, suppressed interfacial thermal accumulation, and ultimately, an augmented surface topography [10]. As proven by Feng et al. [11], the average forces in UVAM were significantly lowered by over 35% compared to conventional milling.

Despite the recognized theoretical merits of UVAM, a comprehensive mechanistic understanding of ultrasonic vibration-assisted milling remains severely bottlenecked by two fundamental research gaps. Firstly, an over-reliance on predictive modeling and a scarcity of empirical physical data. Currently, the prevailing mechanistic analyses regarding friction reduction and kinematic separation in UVM are predominantly derived from Finite Element Method (FEM) simulations or mathematical formulations [12,13]. Constrained by the inherent frequency bandwidth limitations of conventional table-mounted dynamometers (e.g., Kistler) and the complete absence of in-situ sensing capabilities in commercial ultrasonic tool holders, there is a critical void of high-fidelity, high-frequency dynamic mechanical signals acquired directly from the real-world rotating machining interface to validate these simulated models [14,15]. Secondly, the disconnection in cross-scale performance evaluation. Existing literature tends to fragment the cutting force analysis, microstructural evolution, and service performance into isolated studies. While studies have utilized electron backscatter diffraction (EBSD) to observe severe plastic deformation and grain refinement induced by UVAM [16], regarding corrosion resistance, traditional macroscopic electrochemical assessments fundamentally mask the localized electrochemical heterogeneities. For instance, Wang et al. found that UVAM reduced the cutting force, leading the grains to experience elongation, distortion and tilting from shear slip effects [17]. Scant attention has been devoted to unraveling how UVAM-induced textural evolution manipulates the localized micro-galvanic corrosion behavior at the micrometer scale using advanced techniques like the scanning electrochemical microscopy (SECM) method [18].

The main contribution of this work is not to claim that ultrasonic excitation alone determines the corrosion mechanism. Rather, it is to establish and demonstrate a cross-scale experimental framework that simultaneously records machining-load responses at the rotating spindle end and relates them to EBSD-derived subsurface crystallographic features, macroscopic electrochemical parameters, and localized SECM current distributions. Using CM, L-UVAM, and H-UVAM as comparative conditions, the study identifies the experimentally observed associations among load fluctuation, microstructural characteristics, and electrochemical response, while also clarifying the limitations imposed by the absence of independent measurements of surface roughness, residual stress, tool wear, and passive-film chemistry.

## 2. Materials and Methods

### 2.1. Monitoring Setup

All peripheral milling experiments were performed on a CNC machining center (T-600S, Shenzhen Create Century Machinery Co., Ltd., Shenzhen, China). The workpiece material was Ti6Al4V (TC4) alloy (Baoji Titanium technology Co., Ltd., Baoji, China), cut into cubic blocks measuring 40 mm× 40 mm× 40 mm. Milling was conducted using a four-flute solid carbide end mill (D10-C0.1-H25-D10-75L-4F, 10 mm diameter, 0.1 mm corner radius, Shenzhen Multifield Precision Co., Ltd., Shenzhen, China) for all tests.

To continuously capture the dynamic thermo-mechanical responses induced by acoustic vibrations, an innovative cascaded in-situ sensory architecture was constructed. Specifically, a proprietary wireless extrasensory adapter toolholder (iKIT-BT30-BT30-128-H(BCM)-2, IDQ Science and Technology (Hengqin, Guangdong) Co., Ltd., Zhuhai, China), independently developed by our research group, was directly mounted onto the machine spindle. This intelligent module is capable of synchronously monitoring four-dimensional cutting loads—namely cutting force (F), spindle torque (M), and biaxial bending moments (B_1_ and B_2_)—at a sampling frequency of 1024 Hz. This sampling rate was selected to capture the low-frequency and quasi-static components of the machining loads, as well as the load envelope associated with intermittent tool–workpiece engagement. It was not intended to reconstruct the ultrasonic carrier oscillation. Because the ultrasonic operating frequency was in the tens-of-kilohertz range, the carrier waveform itself was not directly resolved by the 1024 Hz data acquisition system. The quantities analyzed in this work, including the mean, peak-to-peak value, RMS value, standard deviation, and coefficient of variation, therefore describe the measured machining-load response and its envelope rather than the instantaneous ultrasonic oscillation. The architectural reliability and sensory fidelity of this wireless toolholder have been comprehensively validated in our previously published studies [19]. Crucially, this smart adapter is equipped with an independent wireless power transmission module (iKIT-O-Charge-E, IDQ Science and Technology (Hengqin, Guangdong) Co., Ltd., Zhuhai, China). As substantiated in our preceding publications, this dedicated power delivery mechanism not only guarantees uninterrupted, long-duration data acquisition but also ensures that the continuous electromagnetic charging process introduces zero parasitic noise, thereby preserving absolute high-fidelity signal stability. Subsequently, the acoustic actuation framework-primarily composed of an external ultrasonic generator, a separate non-contact WPT module dedicated to ultrasonic energy, and the core ultrasonic toolholder (BT30-ER16-N, Shenzhen Multifield Precision Co., Ltd., Shenzhen, China.) was integrated directly beneath the wireless extrasensory adapter toolholder. Functionally, the external generator is responsible for synthesizing electrical signals. By dynamically regulating the driving excitation, it facilitates precise frequency tuning while rigorously sustaining a stable vibrational amplitude at the cutting edge, effectively circumventing the impact of external load fluctuations.

**Figure 1:**
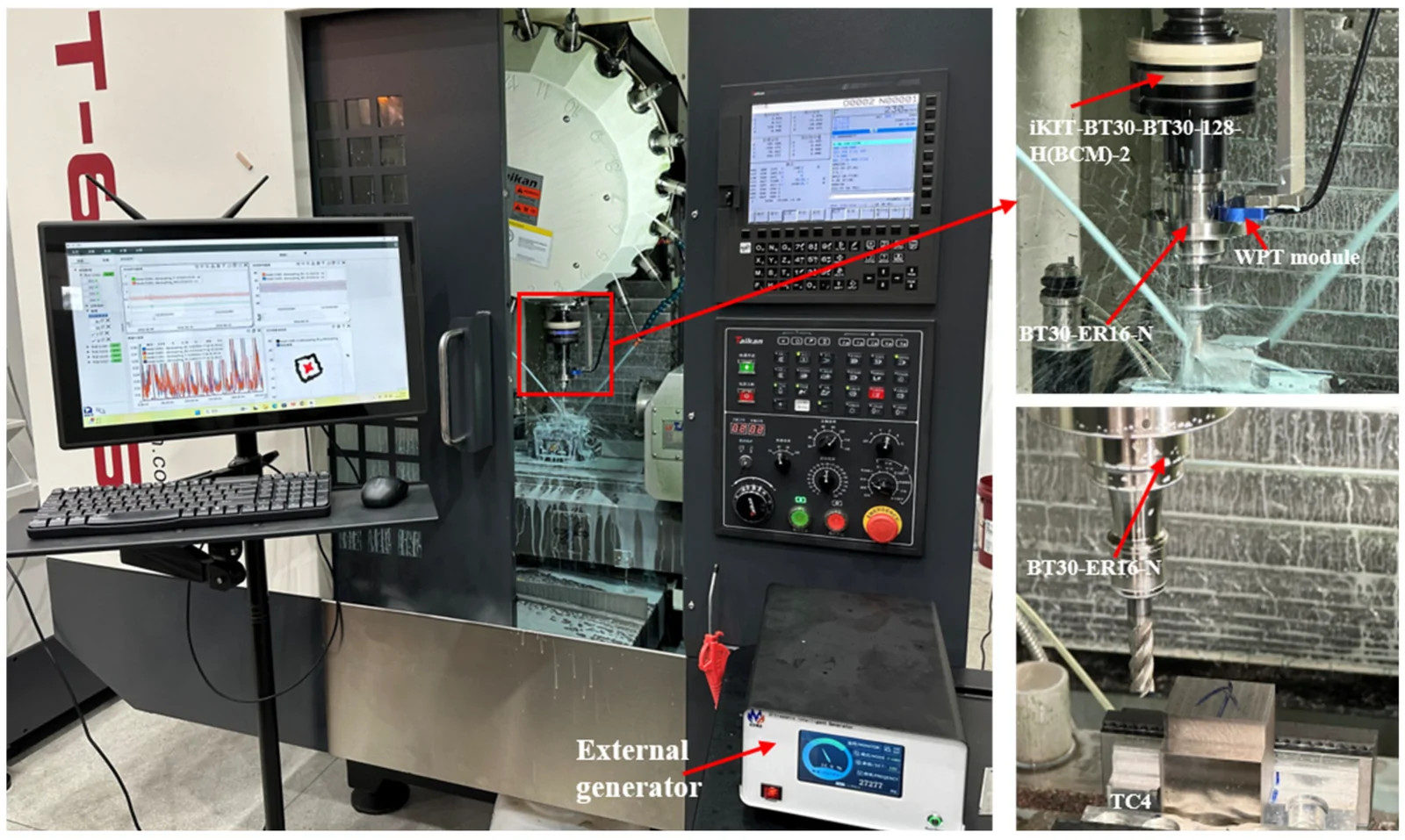
Overall view of the test workspace.

The stable operation of this rotary acoustic system heavily relies on its ultrasonic WPT module, which transfers energy from the stationary machine base to the rotating spindle without physical entanglement. To ensure robust electromagnetic induction coupling and prevent physical collision during high-speed rotation, the spatial clearances were stringently calibrated: the axial standoff distance and the radial concentric gap between the stationary and rotary coils of the ultrasonic holder were precisely adjusted to 0.5 ± 0.05 mm and 0.75 ± 0.2 mm respectively. The detailed physical configuration, encompassing the cascaded dual-WPT adapters, specific assembly gaps, and the localized milling zone, is visually corroborated by the actual experimental photographs presented in Figure 1.

To systematically decode the influence of ultrasonic energy on the machinability of titanium alloys, the experiments were categorized into three distinct kinematic conditions corresponding to three identical workpiece blocks: conventional milling (CM), low-excitation UVAM (L-UVAM, operating at an excitation intensity of 30% of the generator’s capacity) and high-excitation UVAM (H-UVAM, 70% of the maximum generator excitation setting). The cutting parameters were kept strictly constant across all operations to ensure comparability: a spindle speed of 1450 rpm, a feed rate of 230 mm/min, an axial depth of cut ($a_{p}$) of 10 mm, and a radial depth of cut ($a_{e}$) of 0.5 mm. To eliminate the complex engagement variations typically associated with reciprocating toolpaths, a unidirectional down-milling strategy was rigorously adopted. During the operation, the tool traversed along the 40 mm length of the block. Upon completion of a single pass, the cutter was retracted and returned to the origin point. The tool then fed inward by 0.5 mm along the Y-axis, and this down-milling cycle was continuously repeated 20 times per workpiece to generate sufficient machined surface area for subsequent cross-scale characterizations. The same cutting tool was used for the comparative tests, and the machining sequence was kept consistent. However, tool wear was not quantitatively measured using a standardized wear-land parameter in the present study. Consequently, a possible contribution of progressive tool wear to the measured load response and surface condition cannot be completely excluded.

The ultrasonic system was operated in an auto-frequency-tracking mode. The generator excitation levels were set to 30% and 70% for L-UVAM and H-UVAM, respectively. These percentages represent the relative generator setting and should not be interpreted as the actual vibration amplitude. The nominal ultrasonic frequency was 27 kHz, and the corresponding tool-tip displacement amplitude was 0.5 μm. The frequency-tracking system continuously adjusted the excitation frequency to maintain resonance during machining.

### 2.2. Microstructural Characterization

To intuitively unravel the subsurface microstructural degradation and severe plastic deformation mechanisms governed by varying ultrasonic intensities, the cross-sections of the machined specimens (oriented perpendicularly to the feed direction) were meticulously extracted. To eliminate any residual stress layers or micro-scratches induced by mechanical cutting, the cross-sectional surfaces underwent standard mechanical polishing followed by an electrolytic polishing protocol. Metallographic preparation involved sequential mechanical grinding using silicon carbide (SiC) abrasive papers down to 400 grits, followed by multi-stage fine polishing employing monocrystalline diamond suspensions (9 µm, 3 µm, and 1 µm). A final vibratory polishing step with a 500 nm colloidal silica suspension was applied to eradicate residual deformation layers. The microstructural mapping was executed using an Electron Backscatter Diffraction (EBSD) detector (Symmetry S2, Oxford Instruments, Oxford, UK) integrated within a field-emission scanning electron microscope (Sigma 300, Zeiss, Oberkochen, Germany). A scanning step size of 1 μm was selected to ensure statistical reliability across the gradient sub-surface layers. The acquired raw crystallographic data were comprehensively post-processed utilizing Aztec-Crystal software Version 2.2. The raw data were processed using AztecCrystal 2.2. Grains were reconstructed using a minimum misorientation threshold of 5°. Low-angle grain boundaries (LAGBs) were defined as boundaries with misorientations between 2° and 15°, while high-angle grain boundaries (HAGBs) were defined as boundaries with misorientations greater than 15°. The reported grain size corresponds to the equivalent circular diameter calculated from the reconstructed grain area. Grain-boundary fractions were calculated from the total indexed boundary length/area within the mapped region.

### 2.3. Electrochemical Performance

To establish the mapping relationship between surface integrity and anti-corrosion performance, cross-scale electrochemical measurements were performed on the as-machined surfaces at room temperature using a multi-channel electrochemical workstation (Reference 600 Plus, Gamry Instruments, Philadelphia, PA, USA). A standard three-electrode cell configuration was adopted, immersing the samples in a 3.5 wt% NaCl aqueous solution (Shanghai Aladdin Biochemical Technology Co., Ltd., Shanghai, China). A saturated calomel electrode (SCE) and a graphite rod functioned as the reference and counter electrodes, respectively. The samples were encapsulated in epoxy resin, leaving only the unpolished, authentic as-machined surface, with a precisely controlled exposed area of 1 cm^2^, acting as the working electrode. For macroscopic evaluation, the Open Circuit Potential (OCP) was continuously monitored for 3000 s until a stable quasi-steady state was achieved. Subsequently, electrochemical impedance spectroscopy (EIS) was conducted at the OCP, applying a sinusoidal voltage amplitude of 10 mV across a broad frequency sweep from 100 kHz down to 10 mHz. Following EIS, the potentiodynamic polarization (PD) scanning was executed from −500 mV to +500 mV (relative to OCP) at a constant sweep rate of 0.3 mV/s. The EIS spectra were fitted using the equivalent circuit, and the fitted $R_{b}$ value represents the resistance of the barrier-related electrochemical response in the adopted circuit, whereas $n_{b}$ describes the deviation from ideal capacitive behavior. The fitting quality was evaluated using the chi-square value. These parameters should not be interpreted as direct measurements of passive-film thickness or composition.

To evaluate the localized electrochemical reactivity, in-situ scanning electrochemical microscopy (SECM, AMETEK, Inc., Berwyn, PA, USA) was performed utilizing a PAR VersaScan workstation (AMETEK, Inc., Berwyn, PA, USA). The electrochemical circuit was constructed with a classic three-electrode configuration, wherein a 10 μm-diameter platinum (Pt) probe functioned as the dynamic scanning probe. A saturated calomel electrode (SCE) and a high-purity graphite rod were employed as the reference and auxiliary electrodes, respectively.

**Figure 2:**
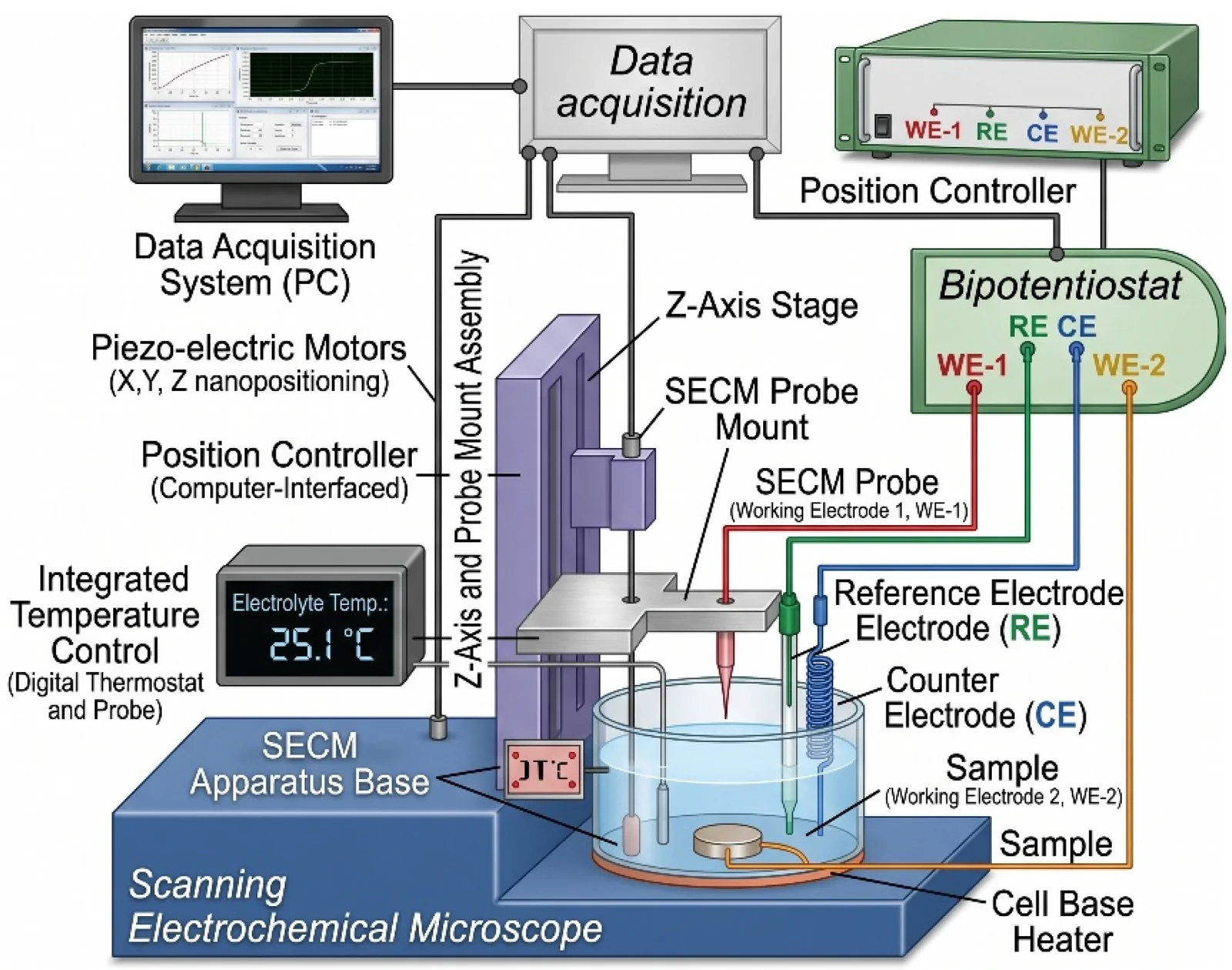
SECM experimental setup diagram.

Thermal equilibrium of the 3.5 wt% NaCl testing fluid was stringently maintained via a digital thermostatic controller (Xiamen Yudian Automation Technology Co., Ltd., Xiamen, China) (Figure 2). To facilitate the feedback mode of the SECM, 2 mM of potassium ferricyanide ($K_{3}[Fe(CN{)}_{6}]$, Shanghai Aladdin Biochemical Technology Co., Ltd. Shanghai, China) was introduced into the electrolyte as the redox mediator. Benefiting from the robust chemical stability and rapid electron-transfer kinetics of the ${\left[Fe(CN{)}_{6}\right]}^{3-}$ species at the Pt interface, the probe was capable of capturing sensitive localized currents [20]. A Pt probe with a nominal diameter of 10 μm was positioned approximately 10 μm above the sample surface based on the approach curve. The scan area was 300 μm × 300 μm and the translation velocity was 15 μm/s. The lateral pixel spacing was 10 μm. During the raster scanning, a continuous anodic bias of +0.51 V (vs. SCE) was polarized on the Pt tip to guarantee the steady-state, diffusion-limited oxidation of the electroactive mediator [21]. The effective electrochemical spatial resolution is governed not only by the pixel spacing but also by the probe diameter, probe–sample separation, feedback mode, and local diffusion field. Accordingly, the SECM maps are interpreted as spatially resolved comparative activity distributions rather than direct maps of individual grains or grain boundaries.

## 3. Results and Discussion

### 3.1. Statistical Characterization of Monitoring Data

**Figure 3:**
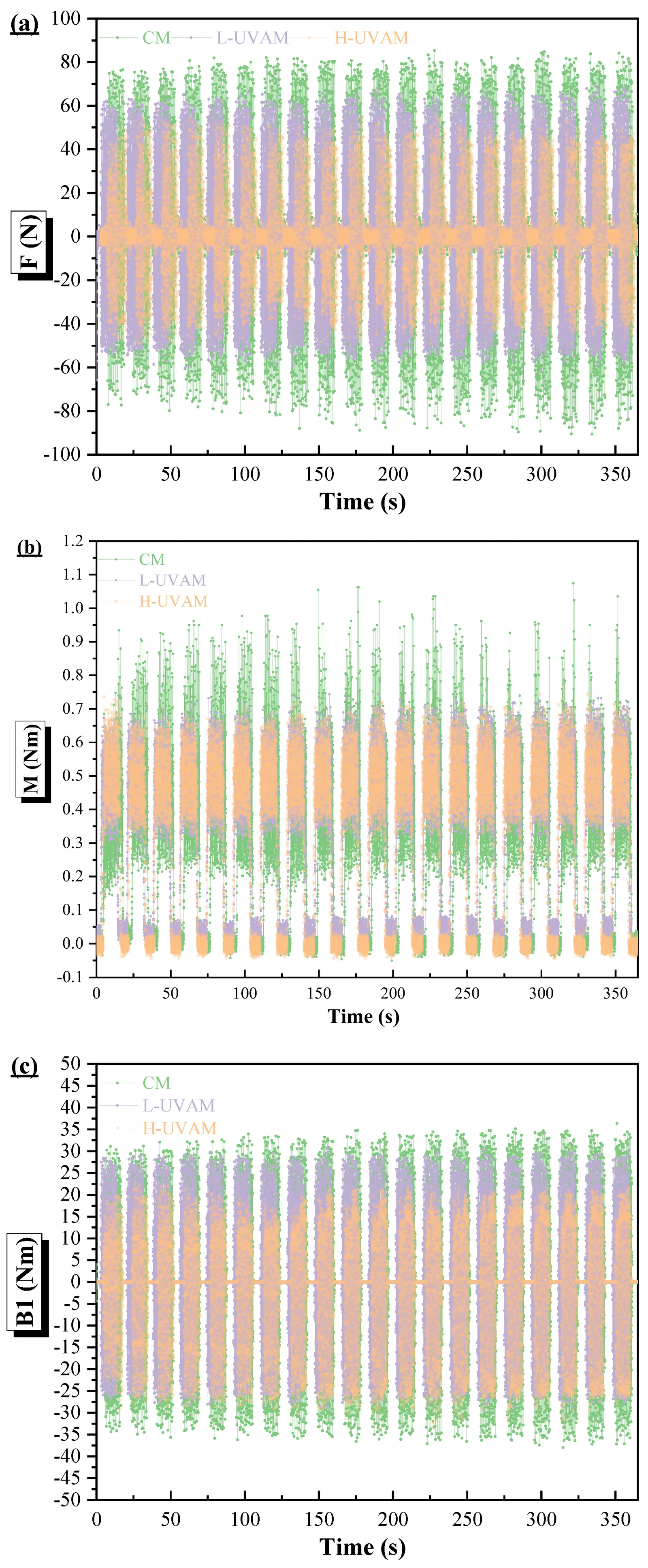

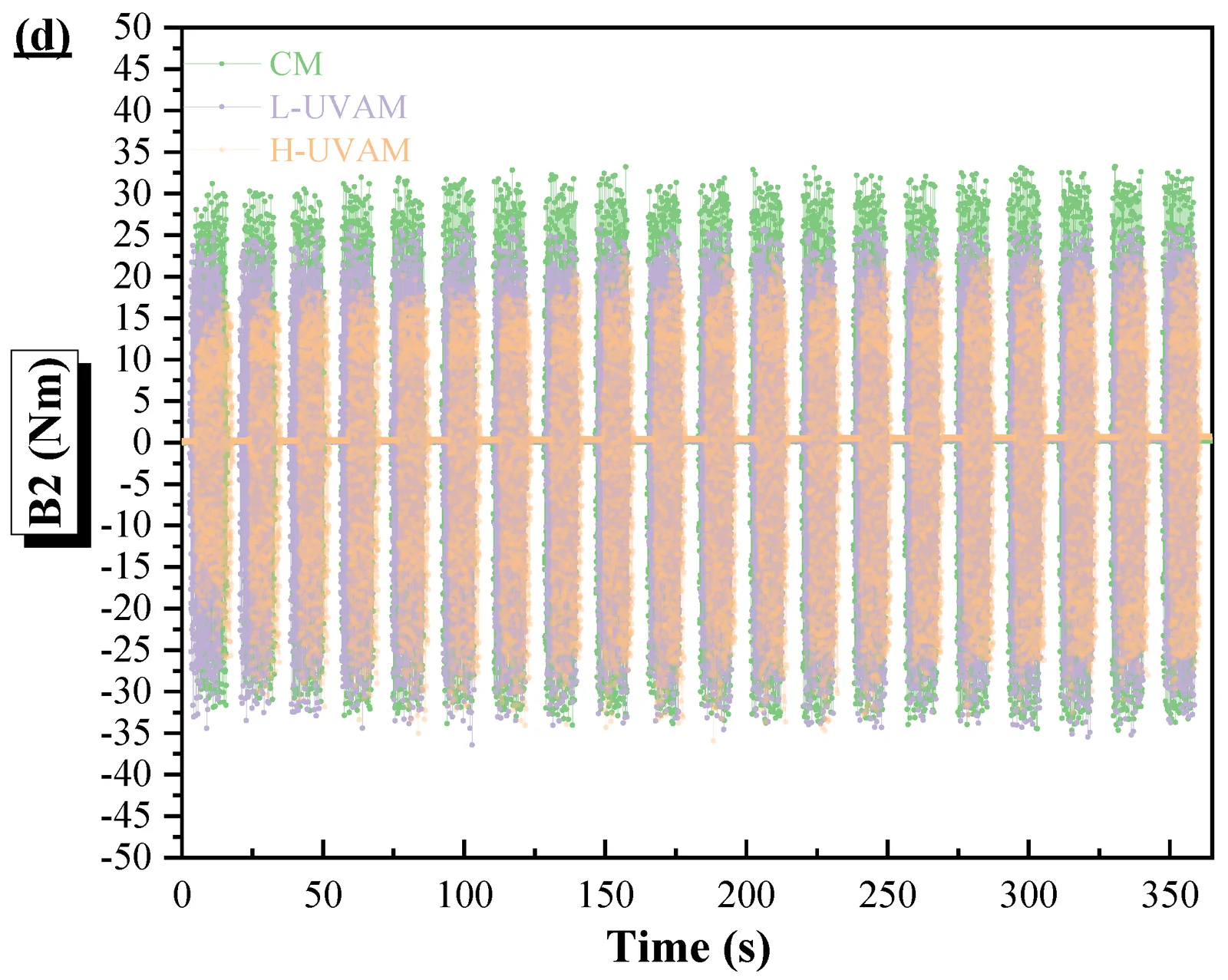
Evolution of the monitoring signals: (**a**) F, (**b**) M, (**c**) B_1_ and (**d**) B_2_.

Figure 3 displays the obtained monitoring data, including F, M, B1 and B2, under three testing conditions. To meticulously deconstruct the perturbing governance of the ultrasonic vibrational field over the non-linear kinetic behavior of the machining ecosystem, a multi-scalar statistical extraction of the high-frequency transient mechanical signals was executed utilizing Origin software (Version 2018). The evaluative matrix incorporates five pivotal statistical descriptors encompassing the arithmetic mean, peak-to-peak excursion, root mean square (RMS), standard deviation (SD), and coefficient of variation (CV). Fundamentally, the RMS metric is mathematically conceptualized to quantify the equivalent continuous energetic dissipation of the non-stationary stochastic signals throughout the cutting regime [22]. It could be calculated through Equation (1), where N denotes the temporal sampling resolution. Meanwhile, the CV functions, as given in Equation (2), as a dimensionless normalized parameter. By filtering out the evaluative bias intrinsically induced by absolute magnitude variations across different kinematic conditions, the CV serves as the quintessential barometer for characterizing the relative dynamic dispersion and anti-chatter robustness of the machining system [23]. All the calculated results are organized in Table 1.

(1) $$RMS=\sqrt{\frac{1}{N}\sum_{i=1}^{N}x_{i}^{2}}$$

(2) CV=(SD/Mean)×100%

The computational derivatives unveil profound mechanical responses across the triad of machining paradigms. Regarding the F, a conspicuous load-alleviation consequence emerges commensurately with the injection and amplification of ultrasonic energy. The F_mean_ progressively deteriorates from 5.67 N in the CM baseline to 4.72 N in the L-UVAM and ultimately to 4.09 N in the H-UVAM. More critically, the suppression of extreme dynamic fluctuations is paramount: under the H-UVAM the F_p-p_ and F_RMS_ plummet aggressively to 70.15 N and 58.72 N, respectively, translating to precipitous reduction of 55.0% and 43.3% relative to the CM condition. The physical genesis of this phenomenon is anchored in the periodic intermittent contact mechanism dictated by the ultrasonic domain [24]. High-frequency oscillations mechanically disrupt the persistent viscous interface between the tool and the workpiece. The curtailed duty cycle not only truncates the effective localized cutting duration but also instigates an acoustic softening phenomenon within the plastic deformation zone via high-frequency micro-impacts, thereby drastically reducing the material’s structural resistance. Furthermore, M_mean_ exhibits profound convergence, residing resiliently within a narrow spectrum of 0.50 to 0.51 Nm across all configurations (Table 1), which validates that the superimposition of ultrasonic kinematics does not compromise the pre-configured volumetric material removal rate. M_p-p_ contracts precipitously from 0.55 Nm (CM) to 0.27 Nm (H-UVAM), elucidating that the ultrasonic perturbation predominantly homogenizes the shearing resistance by ameliorating the tribological distribution on the rake face and optimizing the chip evacuation trajectory, rather than merely mitigating the macroscopic mechanical work.

When analyzing the bending moment, it was found that the p-p bending metrics are elevated under the CM state (B1_p-p_ = 62.85 Nm, B2_p-p_ = 60.79 Nm). This severity originates from consecutive chip agglomeration and sluggish chip evacuation inherent to continuous cutting, which provokes violent asymmetric frictional extrusion between the tool margin and the machined cavity. Following the intervention of high-frequency excitation (H-UVAM), these corresponding spatial vectors regress significantly to 41.76 Nm and 45.00 Nm. The resultant flexural vector ($B_{1-2}$) was computed, and results indicate that H-UVAM not only suppresses the B(1-2)_mean_ from 16.59 Nm (CM) to 11.98 Nm, but critically, it constricts the B(1-2)_SD_ from 13.80 Nm to a mere 9.11 Nm. The high-frequency retreating kinematics of the tool induce a localized hydrodynamic pumping effect, forcefully driving the cutting fluid into the impenetrable core of the cutting zone. This radically transfigures the tribological identity of the tool-chip-workpiece tertiary interface, transitioning from abrasive sliding friction toward elastohydrodynamic lubrication [25]. Concurrently, the cyclic kinetic separation interrupts continuous chip entanglement, preempting tool-center trajectory drift conventionally provoked by the asymmetric proliferation of built-up edges [26]. Electrochemical quantities are reported as mean ± standard deviation based on n = 3 independent measurements.

Furthermore, to definitively assess the intrinsic kinetic stability of the system, the dimensionless coefficient of variation (B(1-2)_CV_) yields conclusive evidence. The B(1-2)_CV_ decreases from 83.20% in the CM condition down to 77.18% (L-UVAM) and reaches 76.08% in the H-UVAM state. This means that the high-frequency ultrasonic energy virtually functions as a kinetic stiffness damper artificially injected into the non-linear machining system, forcefully subjugating low-frequency stochastic chatter, thereby endowing the H-UVAM process with unshakeable operational stability.

### 3.2. EBSD Analysis of Subsurface Microstructures and Crystallography

**Figure 4:**
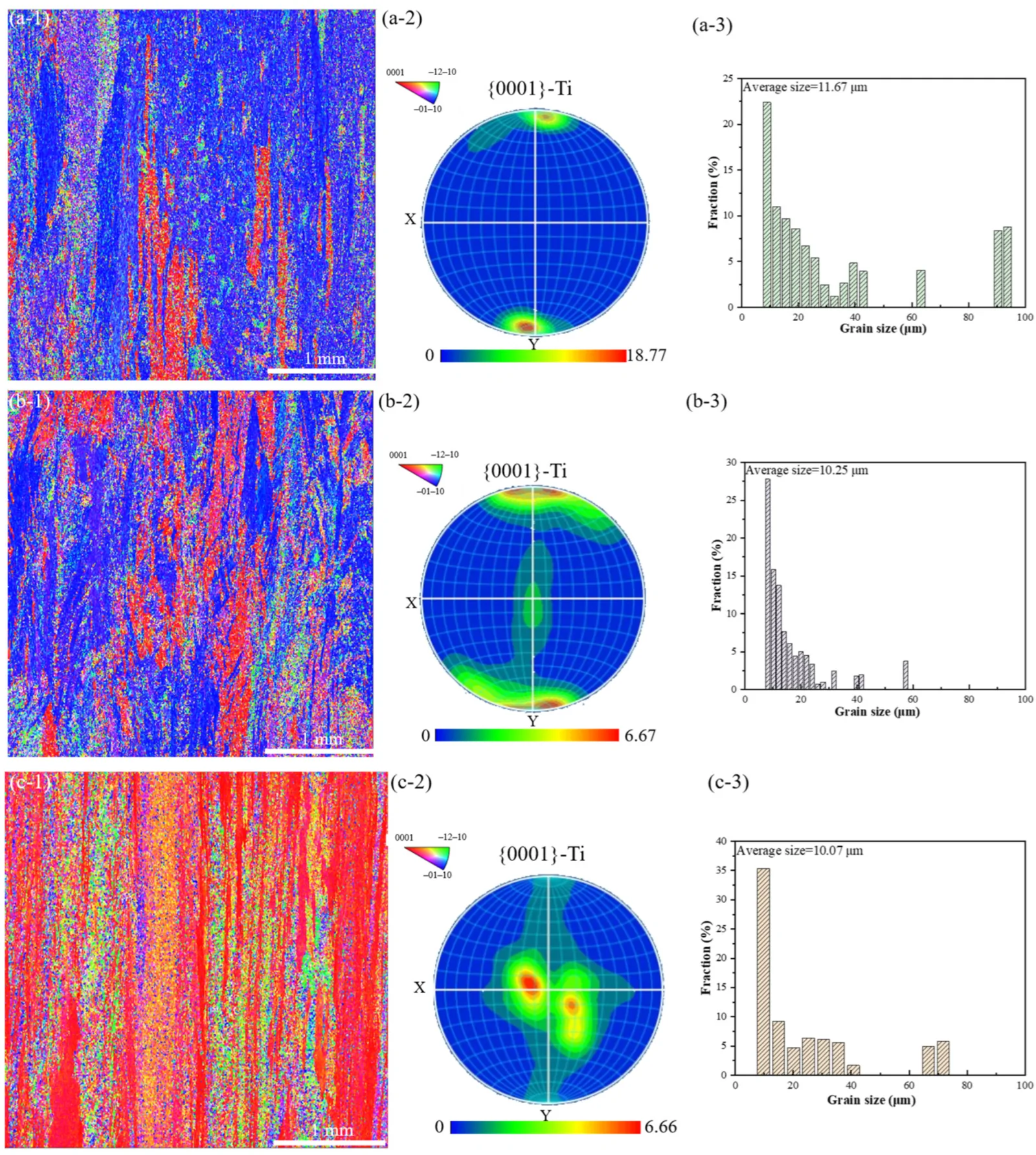
Large-area (3 mm × 3 mm) IPF images of (**a-1**) CM, (**b-1**) L-UVAM, (**c-1**) H-UVAM specimens. The corresponding PF results of (**a-2**) CM, (**b-2**) L-UVAM, (**c-2**) H-UVAM specimens. And grain size distribution plots of the (**a-3**) CM, (**b-3**) L-UVAM, (**c-3**) H-UVAM specimens.

To intrinsically bridge the macroscopic kinetic stabilization unveiled in Section 3.1 with the fundamental surface integrity, EBSD was deployed to map the spatial crystallographic architectures and boundary misorientation distributions beneath the machined sub-surfaces. The large-area (3 mm × 3 mm) inverse pole figure (IPF) topographies, parallelized with the corresponding grain size distributions across the three kinematic paradigms, are comprehensively delineated in Figure 4. Macroscopically, the CM topology exhibits a distinctly heterogeneous micro-morphology, dominated by severely elongated and coarsely deformed grain structures oriented parallel to the cutting direction (Figure 4(a-1)). This morphological distortion is the direct microstructural manifestation of the B1_p-p_ and B2_p-p_ and uninterrupted thermomechanical plowing documented previously. In stark contrast, the superimposition of the high-frequency ultrasonic field fundamentally resculpts the subsurface grain architecture. As statistically synthesized in the histograms (Figure 4(a-3–c-3)), the arithmetic mean grain size undergoes a systematic refinement paradigm, decelerating from 11.67 ± 1.5 μm (CM) to 10.25 ± 1.9 μm in the L-UVAM regime, and ultimately reaching a highly uniform, reduced mean grain size of 10.07 ± 0.9 μm under the H-UVAM state. These values describe the grain population within the mapped regions; because only one workpiece was used for each condition, the comparison should not be interpreted as a fully independent specimen-level statistical test. This progressive micrometre-scale grain refinement is critically driven by the suppression of non-linear mechanical fluctuations. As corroborated by the drastically minimized coefficient of variation (B(1-2)_CV_, Table 1) and converged resultant bending deviations, the high-frequency kinetic stiffness damper essentially eradicates localized stress concentrators and chaotic plowing. Furthermore, the periodic intermittent engagement intrinsically mitigates continuous frictional heat generation, while the acoustically induced pumping effect facilitates deep permeation of the cooling media. This synergistic thermodynamic homogenization eliminates uneven localized thermal gradients, thereby arresting abnormal grain growth and preserving a highly uniform, densely compacted fine-grain layer.

Beyond the morphological miniaturization, the spatially modulated ultrasonic energy drastically transforms the crystallographic orientation anisotropy, as evidenced by the corresponding {0001} pole figures (PFs) in Figure 4. The CM specimen (Figure 4(a-2)) presents an intensely concentrated basal texture, with an overwhelming maximum pole density reaching 18.77. Such a highly polarized crystallographic orientation is typically instigated by the monotonic, unidirectional severe plastic shear strain inherent to conventional continuous turning, which forcibly aligns the basal planes parallel to the dominant shear vector [27,28]. However, the introduction of multi-dimensional ultrasonic perturbations forcefully disrupts this monotonic strain history. The continuous escalation of ultrasonic amplitude dynamically disperses the dense basal poles, culminating in a prominent bimodal and highly weakened texture pattern in the H-UVAM state (Figure 4(c-2)), where the absolute pole density plummets dramatically to 6.66. Mechanistically, this profound texture weakening stems from the acoustic softening phenomenon and the complex triaxial cyclic stress states induced by the ultrasonic impacts. These alternating micro-stresses lower the critical resolved shear stress requisite for dislocation glides, thereby simultaneously activating non-basal slip systems alongside the primary basal slip [29,30]. Consequently, the multidirectional coordinated lattice rotation extensively mitigates crystallographic anisotropy, yielding a more mechanically isotropic subsurface layer that is inherently resilient against micro-crack propagation.

**Figure 5:**
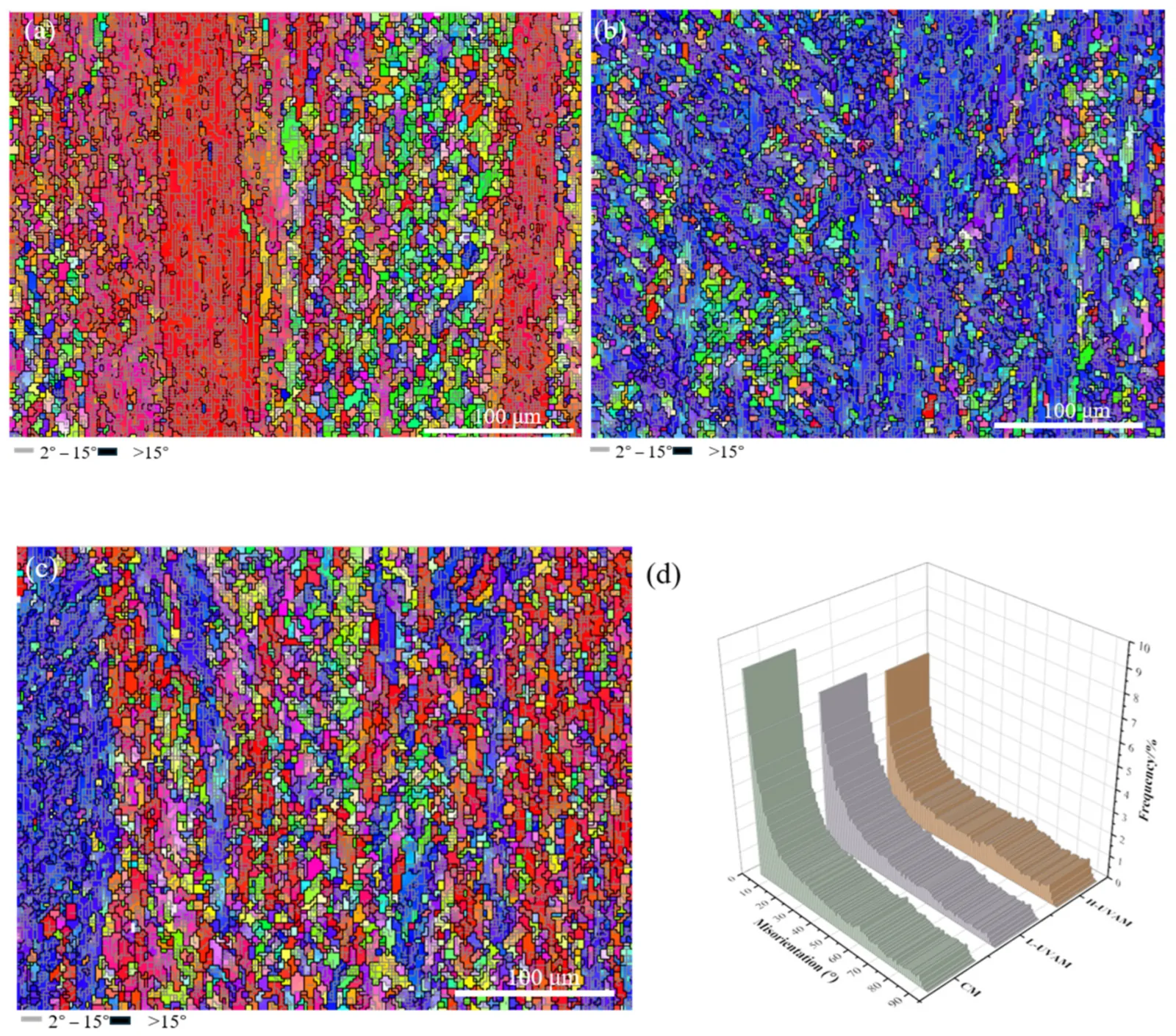
High-magnification IPF maps of the (**a**) CM, (**b**) L-UVAM, and (**c**) H-UVAM specimens; (**d**) comprehensive statistical summary of the crystallographic boundary misorientation fractions across the three kinematic regimes.

To further decode the sub-grain substructural evolution and energy storage characteristics, Figure 5 provides the high-magnification IPF mappings segregating the low-angle grain boundaries (LAGBs, 2–15°, depicted as fine networks) and high-angle grain boundaries (HAGBs, >15°, delineated by bold contours). The statistical boundary misorientation fraction (Figure 5d) exposes a deterministic evolutionary trajectory: the volumetric fraction of HAGBs steadily escalates from a baseline of 48.27% (CM) to 49.24% (L-UVAM), ultimately surging to a remarkable 59.39% in the H-UVAM topology. In physical metallurgy, LAGBs are primarily constituted by thermodynamically unstable dislocation tangles and cellular substructures generated during initial plastic deformation [31]. Under the CM paradigm, the sluggish thermal dissipation and severe spatial moment fluctuations promote profound dislocation entanglements without sufficient driving force for structural recovery. Conversely, the high-frequency cyclic impacts inherent to H-UVAM inject a massive quantum of cumulative strain energy into the lattice matrix in a spatially uniform manner. This excessive local stored energy, coupled with localized adiabatic heating within the microscopic shear bands, may facilitate dynamic recovery and progressive subgrain rotation. The increase in the measured HAGB fraction is consistent with the conversion of some low-angle substructures into higher-misorientation boundaries; however, the present EBSD data alone are insufficient to conclusively identify continuous dynamic recrystallization [32]. As a result, the pre-existing dense dislocation walls continuously absorb dislocations and undergo progressive sub-grain rotation, suggesting a redistribution of boundary misorientations from low-angle substructures toward a higher fraction of HAGBs [33]. Crucially, this augmented proliferation of stable HAGBs constructs an ultra-dense array of crystalline interfaces. As will be systematically diagnosed in the subsequent electrochemical impedance spectroscopy (EIS) evaluations, these dense boundary networks serve as highly active thermodynamic nucleation sites, thereby massively accelerating the spontaneous formation of an impervious, protective passive oxide film when exposed to corrosive environments.

### 3.3. Electrochemical Degradation Behavior

**Figure 6:**
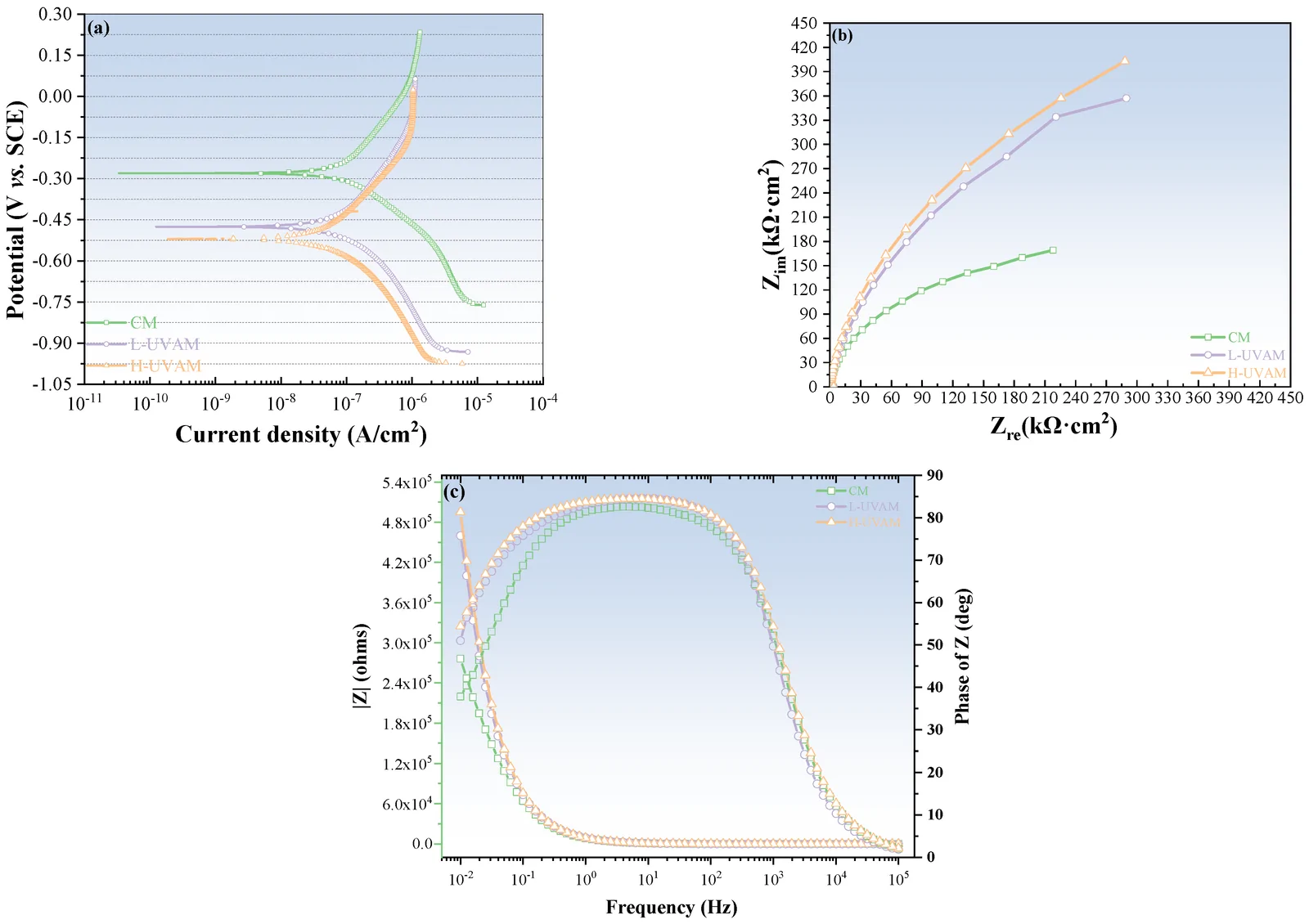
Electrochemical response of the CM, L-UVAM, and H-UVAM specimens: (**a**) potentiodynamic polarization curves, (**b**) Nyquist plots, (**c**) Bode magnitude plots and Bode phase-angle plots.

To elucidate the intrinsic influence of high-frequency ultrasonic mechanical perturbations on the environmental serviceability of the machined topologies, the macroscopic electrochemical kinetic signatures across the three kinematics were systematically quantified. Figure 6 delineates the PD trajectories alongside the Nyquist and Bode impedance spectra, with the corresponding equivalent circuit (EEC) variables extracted in Table 2. The PD curves (Figure 6a) comprehensively map the thermodynamic corrosion propensity and kinetic dissolution velocity. As delineated in Table 2, the superimposition of ultrasonic vibrational energy instigates a conspicuous cathodic shift in the free corrosion potential (E_corr_), migrating progressively from −273.14 ± 25.24 mV in CM condition to −517.32 ± 9.04 mV in the H-UVAM condition. The negative shift reflects a change in the mixed potential of the surface and is not interpreted independently as evidence of higher surface energy [34]. Mechanistically, the severe plastic deformation (SPD) and high-frequency micro-impacts inherent to H-UVAM introduce dense crystalline defects, which act as active energetic sites. Interestingly, this thermodynamic activation is highly beneficial for passivating alloys. The elevated surface energy facilitates the rapid nucleation and spontaneous precipitation of surface oxides [35]. Consequently, this rapid passivation fundamentally suppresses the actual corrosion kinetics. The corrosion current density (I_corr_), which serves as the direct kinetic barometer for the dissolution rate, manifests a dramatic attenuation. The I_corr_ of the H-UVAM topological matrix decelerates precipitously to merely 0.022 ± 0.005 μA·cm^−2^, constituting an approximate 65.6% reduction relative to the CM regime (0.064 ± 0.004 μA·cm^−2^). The physical genesis of this suppressed kinetic dissolution rate is directly traceable to the spatial dynamic instability suppression mechanism authenticated in Section 3.1. Under CM, violent flexural moment fluctuations engrave severe mechanical plowing traces and stress concentrators, which act as micro-anodic sites driving galvanic corrosion. Conversely, the kinetic stiffness damper in H-UVAM drastically mitigates these topological physical defects, choking the pathways for localized active dissolution [36].

To further decipher the interfacial charge transfer kinetics and the spatial compactness of the passive barrier, EIS yields conclusive diagnostic evidence. In the Nyquist complex plane (Figure 6b), the capacitive arc radius is directly proportional to the physical resistance of the surface barrier against corrosive medium permeation. Manifestly, the capacitive arc of H-UVAM comprehensively eclipses those of CM and L-UVAM, epitomizing unparalleled interfacial polarization resistance. Coupled with the EEC optimizations in Table 2, the barrier layer resistance (R_b_) under H-UVAM escalates substantially to (8.89 ± 0.41) × 10^4^ Ω·cm^2^, which is approximately 2.58 times higher than that of the CM counterpart ((3.44 ± 0.19) × 10^4^ Ω·cm^2^). Notably, the dispersion exponent (n_b_), which diagnoses the non-ideal capacitive behavior induced by surface heterogeneity, increases from 0.91 (CM) to 0.94 (H-UVAM), trending toward unity. The increase in $R_{b}$ and $n_{b}$ indicates a higher fitted resistance and a less dispersive capacitive response of the H-UVAM surface. These changes are consistent with a more protective and electrochemically homogeneous surface condition. However, EIS alone cannot determine the chemical composition, thickness, or compactness of the passive film, and the present measurements cannot separate the effects of microstructure, surface roughness, residual stress, and other surface-layer characteristics. Therefore, the EIS results are interpreted here as evidence of improved interfacial electrochemical behavior rather than direct proof of passive-film densification. Bridging this to the preceding monitoring results (Section 3.1), the rigorous structural uniformity engineered by stabilized cutting forces strictly averts micro-cracks or intergranular tearing. This homogeneous cold-shearing phenomenon ensures the defect-free, two-dimensional continuous proliferation of the superficial oxide scale, thereby forging an impregnable electrochemical defense fortress [37].

However, it should be noted that surface roughness, residual stress, near-surface hardness, and passive-film chemistry were not independently measured in this study. These factors may contribute to the observed electrochemical differences. Therefore, the present results establish a correlation among machining-load fluctuations, crystallographic characteristics, and electrochemical response, but they do not isolate the individual contribution of each surface-integrity parameter.

### 3.4. SECM Topography and Localized Reactivity

**Figure 7:**
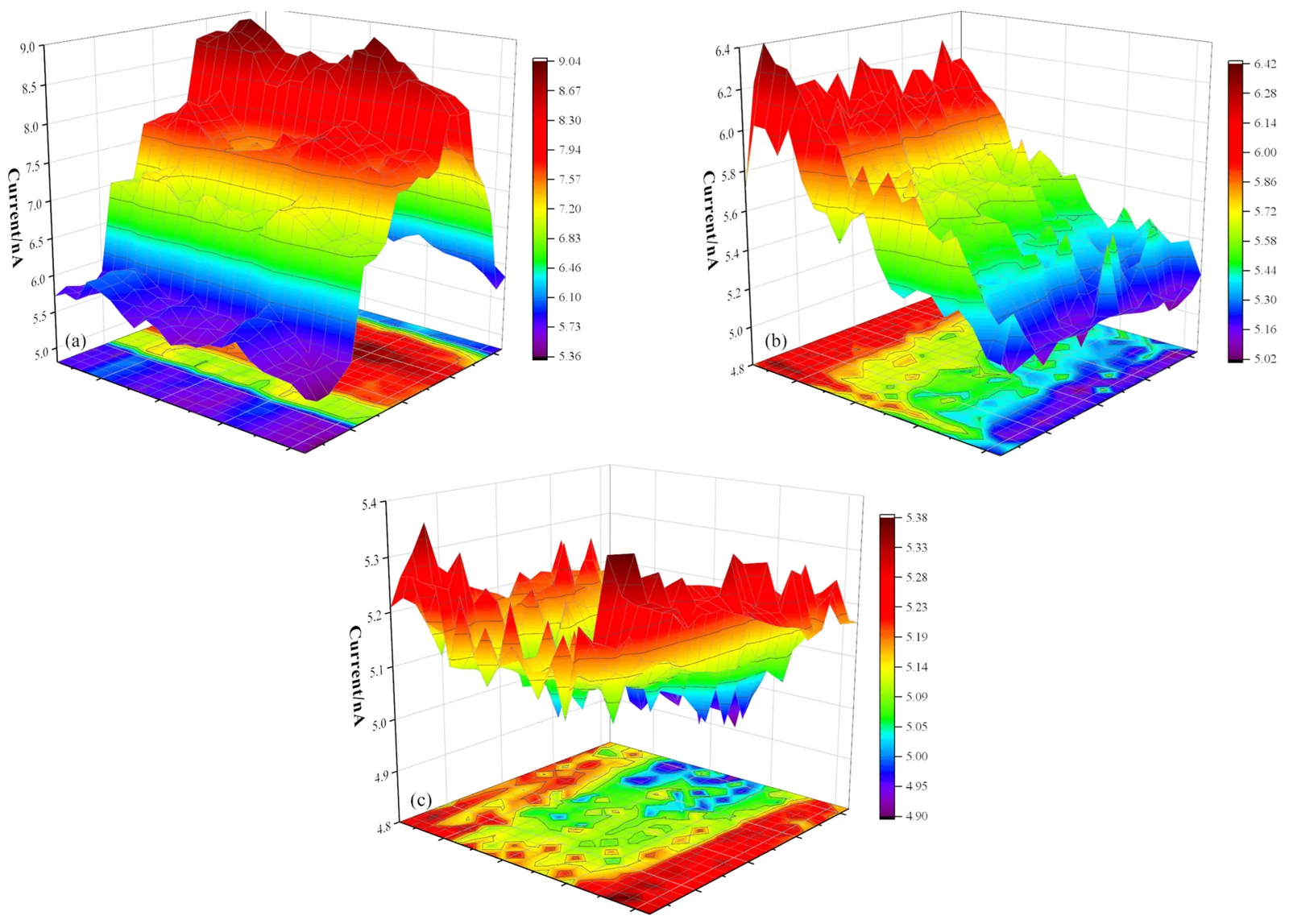
SECM results of the (**a**) CM, (**b**) L-UVAM, and (**c**) H-UVAM specimens.

SECM was used to compare the spatial distribution of electrochemical activity under identical mediator, probe-potential, probe-height, and scanning conditions. The measured probe current reflects the local feedback response of the redox mediator and is influenced by the local interfacial electrochemical environment. Therefore, the SECM current should be interpreted as a comparative indicator of spatial electrochemical heterogeneity rather than as a direct measurement of the local corrosion rate. Figure 7 visualizes the highly localized electrochemical activity across the machined sub-surfaces via 3D triangulated current distribution topographies coupled with 2D heat maps. CM specimen (Figure 7a) exhibits a distinctly heterogeneous micro-electrochemical topography, characterized by a high average anodic current of 6.83 nA and a massive spatial current fluctuation ranging from 5.36 to 9.04 nA. This severe localized current gradient visually manifests as steep topographic peaks and concentrated red zones in the corresponding heat map, signifying highly localized anodic dissolution and an unstable passive state. Conversely, the continuous escalation of ultrasonic energy fundamentally pacifies the surface reactivity. For the L-UVAM state (Figure 7b), the mean current decays to 5.60 nA with a narrowed gradient (5.02–6.42 nA). Most strikingly, the H-UVAM regime (Figure 7c) yields an exceptionally flat and globally homogenized electrochemical response, where the mean current plummets to a mere 5.14 nA and the spatial fluctuation is tightly constrained within an ultra-narrow band of 4.90 to 5.38 nA, thoroughly eliminating the prominent localized active spots observed in the CM counterpart.

This profound electrochemical homogenization observed in the H-UVAM state is not an isolated phenomenon but is intrinsically governed by the crystallographic architecture previously elucidated in Section 3.2. In physical electrochemistry, vast spatial current gradients, such as those in the CM state, are typically driven by the formation of intensive micro-galvanic cells. Under machining, the heterogeneous coarse grains, intense basal texture polarization, and highly localized residual stress concentrations collectively create micro-regions with varying electrochemical potentials [38]. The thermodynamically unstable LAGBs and heavily deformed zones act as preferential anodic dissolution sites, thereby resulting in the rugged SECM current topography. In stark contrast, the highly uniform, micro-refined grain structure and the extensively weakened basal texture induced by H-UVAM essentially eradicate these structural anisotropies. Crucially, the massive proliferation of thermodynamically stable High-Angle Grain Boundaries (HAGBs, surging to 59.39% as quantified in Figure 5) constructs a hyper-dense network of electrochemical nucleation sites. During initial exposure to the corrosive medium, these abundant HAGBs accelerate the solid-state diffusion of passivating elements, promoting the instantaneous and spontaneous formation of a highly compact, dense, and continuous passive oxide film [39]. This superior passive layer physically shields the substrate, translating macroscopically into the ultra-low and uniformly distributed SECM current profile.

Tracing the causality back to the initial macroscopic kinematic paradigms monitored in Section 3.1, the genesis of this exceptional electrochemical integrity is deeply rooted in the suppression of non-linear thermomechanical fluctuations. The high-frequency ultrasonic kinetic stiffness damper effectively mitigates chaotic plowing and uneven frictional heat generation, ensuring that the cumulative strain energy and thermal distribution injected into the subsurface are spatially uniform. This thermodynamic homogenization arrests abnormal localized defect accumulation, ultimately guaranteeing the formation of a homogeneous crystalline matrix. Consequently, this multi-scale structural-functional mapping explicitly demonstrates that the ultrasonic-driven kinetic stabilization at the macro-scale translates directly into microstructural refinement, which in turn seamlessly dictates the superior and uniform electrochemical corrosion resistance during actual service.

## 4. Conclusions

In this study, a wireless in-situ monitoring system was combined with EBSD, potentiodynamic polarization, EIS, and SECM to compare conventional milling, low-excitation UVAM, and high-excitation UVAM of Ti-6Al-4V. Within the scope of the tested machining conditions, the following conclusions can be drawn:

1. The wireless monitoring system recorded the axial load component, spindle torque, and two bending-moment components at 1024 Hz. The analyzed signals represent the low-frequency machining-load response and load envelope, rather than the ultrasonic carrier oscillation itself. Under H-UVAM, the RMS value of the measured axial load component decreased by 43.3% compared with CM, while the variation in the resultant bending-moment signal was also reduced.
2. EBSD characterization showed a decrease in the mean equivalent grain size from 11.67 μm under CM to 10.07 μm under H-UVAM. The measured HAGB fraction increased from 48.27% to 59.39%, and the basal texture intensity was reduced. These observations indicate that UVAM modified the subsurface crystallographic state under the tested conditions. However, the present EBSD results do not independently prove dynamic recrystallization or identify the individual causes of grain refinement.
3. The H-UVAM surface exhibited a lower measured corrosion current density and a higher fitted barrier resistance than the CM surface. The negative shift in $E_{corr}$ was interpreted only as a change in mixed electrochemical potential and was not used independently as an indicator of corrosion resistance or surface energy. The EIS parameters indicate a changed barrier-related electrochemical response but do not directly establish passive-film composition, thickness, or densification.
4. SECM measurements showed that the H-UVAM condition produced a narrower local probe-current distribution than CM under identical measurement conditions, indicating a more spatially uniform electrochemical response. The SECM current was interpreted as a comparative indicator of local electrochemical activity rather than a direct local corrosion-rate measurement.
5. Overall, the results support an association among reduced machining-load fluctuation, modified subsurface crystallographic features, and improved electrochemical response. Nevertheless, surface roughness, residual stress, tool wear, passive-film chemistry, and independent workpiece-level variability were not fully quantified in the present study. These factors should be investigated in future work to separate their respective contributions.

## Figures and Tables

**Table 1:** Statistical feature comparison of the monitoring signals.

	CM	L-UVAM	H-UVAM
F_mean_/N	5.67 ± 0.48	4.72 ± 0.37	4.09 ± 0.41
F_p-p_/N	156.05 ± 14.17	113.01 ± 13.40	70.15 ± 8.73
F_RMS_/N	103.53 ± 10.87	95.31 ± 14.30	58.72 ± 9.33
M_mean_/Nm	0.50 ± 0.04	0.51 ± 0.05	0.50 ± 0.04
M_p-p_/Nm	0.55 ± 0.05	0.30 ± 0.04	0.27 ± 0.03
M_RMS_/Nm	1.18 ± 0.11	1.24 ± 0.17	1.26 ± 0.13
B1_p-p_/Nm	62.85 ± 7.25	52.32 ± 6.77	41.76 ± 3.91
B2_p-p_/Nm	60.79 ± 9.17	55.25 ± 8.10	45.00 ± 5.10
B(1-2)_mean_/Nm	16.59 ± 1.67	15.27 ± 1.17	11.98 ± 1.80
B(1-2)_SD_/Nm	13.80 ± 2.20	11.79 ± 1.88	9.11 ± 0.93
B(1-2)_RMS_/Nm	68.23 ± 10.11	60.99 ± 8.28	48.61 ± 6.20
B(1-2)_CV_/%	83.20 ± 8.47	77.18 ± 8.22	76.08 ± 9.41

**Table 2:** Corrosion parameters of the test specimens.

	CM	L-UVAM	H-UVAM
E_corr_/mV	−273.14 ± 25.24	−477.81 ± 5.35	−517.32 ± 9.04
I_corr_/μA·cm^−2^	0.064 ± 0.004	0.041 ± 0.003	0.022 ± 0.005
R_s_/Ω·cm^2^	8.95 ± 0.69	9.70 ± 0.76	7.90 ± 0.69
Q_b_/10^−7^ F/cm^2^	2.31 ± 0.18	2.05 ± 0.22	2.03 ± 0.11
R_b_/10^4^ Ω·cm^2^	3.44 ± 0.19	7.58 ± 0.63	8.89 ± 0.41
n_b_	0.91	0.93	0.94
λ	1.47 × 10^−3^	1.50 × 10^−3^	7.86 × 10^−4^

## Data Availability

The original contributions presented in this study are included in the article. Further inquiries can be directed to the corresponding authors.
